# Supplementary material for: CircEHD2, CircNETO2 and CircEGLN3 as Diagnostic and Prognostic Biomarkers for Patients with Renal Cell Carcinoma
Source: Cancers (Basel). 2021 Apr 30;13(9):2177. doi: 10.3390/cancers13092177 (PMC8124893; doi:10.3390/cancers13092177)
Supplement: Supplementary file 1 [file cancers-13-02177-s001.zip › cancers-1132882-supplementary.pdf]

cancers MDPI

Article

Supplementary Information: CircEHD2, circNETO2 and circEGLN3 as diagnostic and prognostic biomarkers for patients with renal cell carcinoma

Lisa Frey, Niklas Klümper, Doris Schmidt, Glen Kristiansen, Marieta Toma, Manuel Ritter, Abdullah Alajati and Jörg Ellinger

Primer Design:

Divergent circRNA-specific PCR primers for circEHD2, circCOL5A1, circNETO2 and circSCARB1 were self-designed using the CircInteractome tool. Details on the primer design are provided below. The primer binding sites are indicated by arrows (>>> fwd-primer, <<< rev-primer), the last/first 100 bp at the junction site are indicated in blue/red. All primers were specific for the specified circRNA splice variant.

circEHD2

**CircRNA ID:**	hsa_circ_0003146
**Location:**	chr19:48229068-48229481
**PCR product length:**	103 bp
**Forward primer:**	5′-CTG-GTG-CGA-GCT-ACG-ACT-TC-3′
**Reverse primer:**	5′-TCG-TCC-GAG-ATC-TCC-AGC-TT-3′),
**Junction sequence (3′End-5′End):**	Exon4 – Exon4

GCTGGAGGAGCAGGACCTCTTCCGCGACATCCAGGGCCTGCCCCGGCACGCAGCCTTGCG-
CAAGCTCAACGACCTGGTGAAGAGGGCCCGGCTGGTGCGA
>>>>>>>>>
GCTACGACTTCCCGGCCGTGCTGCGCTGGTTCGCGGAGCGCGTGGAC-
CTCATCATCCTGCTCTTTGATGCGCACAAGCTGGAGATCTCGGACGAGTTCTC
>>>>>>>>>>> <<<<<<<<<<<<<<<<<<<<

circCOL5A1

**CircRNA ID:**	hsa_circ_0003596
**Location:**	chr9:137716445-137717750
**PCR product length:**	95 bp
**Junction sequence (3′End-5′End):**	

CGATCCTAACCAAGGATGCTCCAGGGATTCCTTCAAGGTTTACTG-
CAACTTCACAGCCGGGGGGTCGACATGCGTCTTCCCTGACAAGAAGTCCGAAGGG
>>>>>>>>>>>>>>>>>>>> <<<<<<<<<<<<<<
GGCCCCCCGGGAGAGGTCATCCAGCCCCTGCCAATCCAGGCATCCAGGACGCGGCG-
GAACATCGACGCCAGCCAGCTGCTGGACGACGGGAATGGCGAGA
<<<<

circNETO2

**CircRNA ID:**	hsa_circ_0003520
**Location:**	chr16:47143393-47165936
**PCR product length:**	84 bp
**Junction sequence (3′End-5′End):**	Exon7 – Exon2

GATGTAATGCTTAAAACAGGAATTGGAGTGATTCGAATGTGGGCAGATGAAGGTAG-
TCGGCTTAGCAGGTTTCGAATGCTCTTTACTTCCTTTGTGGAGC
>>>>>>>>>>>>>>>>>>>>> <<<<<<<<<<<<<
TGTTGTTAATAACAGTACTGGTAGTGGAAGGGATTGCCGTGGCCCAAAAAACCCAA-
GATGGACAAAATATTGGAATCAAGCATATTCCTGCAACCCAGTG
<<<<<<<<<<

<u>circSCARB1</u>

CircRNA ID: hsa_circ_0029340
Location: <u>chr12:12529230-125294835</u>
PCR product length: 88 bp
Junction sequence (3′End-5′End): Exon7 – Exon6

CCCAAAACCCTGTTTGCCAACGGGTCCATCTACCCACCCAAC-
GAAGGCTTCTGCCCGTGCCTGGAGTCTGGAATTCAGAACGTCAGCACCTGCAGGTTCA
>>>>>>>

GTTGACTTCTGGCATTCCGATCAGTGCAACATGATCAATGGAACTTCTGGG-
CAAATGTGGCCGCCCTTCATGACTCCTGAGTCCTCGCTGGAGTTCTACA
>>>>>>>>>>>>>> <<<<<<<<<<<<<<<<<<<<

RT-qPCR methodology - MIQE checklist according to Bustin et al.

RT-qPCR measurements were performed according to the recommendations of the MIQE guidelines. Both no template controls (NTC) and no reverse transcription controls (No-RT controls) were performed and showed always negative results.

Item to Check	Importance	Checklist	Where; Comment
		**Experimental Design**	
Definition of experimental and control groups	**E**	Yes	Main text: Materials and Methods; Results: Table 1
Number within each group	**E**	Yes	Main text: Materials and Methods; Results: Table 1
Assay carried out by core lab or investigator's lab?	D	Yes	Investigator's lab
Acknowledgement of authors' contributions	D	Yes	Main text
		**Sample**	
Description	**E**	Yes	Main text: Results; Materials and Methods.
Volume/mass of sample processed	D	Yes	Main text: Results; Materials and Methods
Microdissection or macrodissection	**E**	Yes	microdissection
Processing procedure	**E**	Yes	Main text: Results; Materials and Methods
If frozen - how and how quickly?	**E**	Yes	Snap-frozen
If fixed - with what, how quickly?	**E**	No	
Sample storage conditions and duration (esp. for FFPE samples)	**E**	Yes	Main text: Materials and Methods
		**Nucleic Acid Extraction**	
Procedure and/or instrumentation	**E**	Yes	Main text: Materials and Methods
Name of kit and details of any modifications	**E**	Yes	Main text: Materials and Methods
Source of additional reagents used	D	Yes	Main text: Materials and Methods
Details of DNase or RNase treatment	**E**	Yes	Main text: Materials and Methods
Contamination assessment (DNA or RNA)	**E**	Yes	genomic DNA contamination was excluded by control experiments without reverse transcription
Nucleic acid quantification	**E**	Yes	Main text: Materials and Methods
Instrument and method	**E**	Yes	Main text: Materials and Methods
Purity (A260/A280)	D	Yes	Nanodrop 2000
Yield	D	Yes	Nanodrop 2000 (1,7 – 2,0)
RNA integrity method/instrument	**E**	Yes	Electrophoresis
RIN/RQI or Cq of 3' and 5' transcripts	**E**	No	
Electrophoresis traces	D	Yes	

Inhibition testing (Cq dilutions, spike or other)	**E**	No	
		**Reverse Transcription**	
Complete reaction conditions	**E**	Yes	Main text: Material and Methods
Amount of RNA and reaction volume	**E**	Yes	Main text: Material and Methods
Priming oligonucleotide (if using GSP) and concentration	**E**	Yes	Main text: Material and Methods
Reverse transcriptase and concentration	**E**	Yes	Main text: Material and Methods
Temperature and time	**E**	Yes	Main text: Material and Methods
Manufacturer of reagents and catalogue numbers	D	Yes	Main text: Material and Methods
Cqs with and without RT	D	Yes	methodology: negative results
Storage conditions of cDNA	D	Yes	Main text. Materials and Methods; storage at -20°C
		**qPCR Target Information**	
Gene symbols	**E**	Yes	Main text: Materials and Methods
If multiplex, efficiency and LOD of each assay.	**E**	No	Not applicable
Sequence accession number	**E**	Yes	Main text: has_circ_nr.
Location of amplicon	D	Yes	Main text: Materials and Methods
Amplicon length	**E**	Yes	Main text: Materials and Methods
In silico specificity screen (BLAST, etc)	**E**	Yes	Main text: Materials and Methods
Pseudogenes, retropseudogenes or other homologs?	D	No	Not applicable
Sequence alignment	D	Yes	Main text: Materials and Methods
Secondary structure analysis of amplicon	D	No	Not applicable
Location of each primer by exon or intron (if applicable)	**E**	Yes	Supplementary Information S1: the self-designed primers by Circinteractome
What splice variants are targeted?	**E**	Yes	only one splice variant
		**qPCR Oligonucleotides**	
Primer sequences	**E**	Yes	Main text: Materials and Methods (self-designed primers by Circinteractome)
RTPrimerDB Identification Number	D	No	Not applicable
Probe sequences	D	No	Not applicable
Location and identity of any modifications	**E**	No	Not applicable
Manufacturer of oligonucleotides	D	Yes	Thermo Fisher Scientific
Purification method	D	Yes	desalted
		**qPCR Protocol**	
Complete reaction conditions	**E**	Yes	Main text: Materials and Methods.
Reaction volume and amount of cDNA/DNA	**E**	Yes	Main text: Materials and Methods.
Primer, (probe), Mg++ and dNTP concentrations	**E**	Yes	Main text: Materials and Methods.
Polymerase identity and concentration	**E**	Yes	Main text: Materials and Methods.
Buffer/kit identity and manufacturer	**E**	Yes	Main text: Materials and Methods.
Exact chemical constitution of the buffer	D	Yes	Kit Takara Nr. RR037A

Additives (SYBR Green I, DMSO, etc.)	**E**	Yes	Main text: Materials and Methods.
Manufacturer of plates/tubes and catalogue number	D	Yes	384 Well PCR plate (Thermo Fisher 732-4961)
Complete thermocycling parameters	**E**	Yes	Main text: Materials and Methods
Reaction setup (manual/robotic)	D	Yes	Manual setup
Manufacturer of qPCR instrument	**E**	Yes	Main text: Materials and Methods: QuantStudio 5 Real-Time PCR System; Applied Biosystems; Thermo Fisher Scientific
		**qPCR Validation**	
Evidence of optimisation	D	Yes	Melt Curve Plot analysis
Specificity (gel, sequence, melt, or digest)	**E**	Yes	Gel electrophoresis
For SYBR Green I, Cq of the NTC	**E**	Yes	undetermined
Standard curves with slope and y-intercept	**E**	Yes	Calculated by QS5 software
PCR efficiency calculated from slope	**E**	Yes	Main text: Materials and Methods.
Confidence interval for PCR efficiency or standard error	D	Yes	95
r2 of standard curve	**E**	Yes	Calculated by QS5 software
Linear dynamic range	**E**	Yes	Standard curve dilution
Cq variation at lower limit	**E**	Yes	
Confidence intervals throughout range	D	No	
Evidence for limit of detection	**E**	No	
If multiplex, efficiency and LOD of each assay.	**E**	Not applicable	
		**Data Analysis**	
qPCR analysis program (source, version)	**E**	Yes	Main text: Materials and Methods (ThermoFisher Cloud 2.0)
Cq method determination	**E**	Yes	QS5 standard analysis settings
Outlier identification and disposition	**E**	Not applicable	
Results of NTCs	**E**	Yes	Main text: Materials and Methods.
Justification of number and choice of reference genes	**E**	Yes	Main text: Materials and Methods.
Description of normalisation method	**E**	Yes	Main text: Materials and Methods.
Number and concordance of biological replicates	D	No	Not applicable
Number and stage (RT or qPCR) of technical replicates	**E**	Yes	triplicates
Repeatability (intra-assay variation)	**E**	Yes	Inter-calibration settings -> positive control
Reproducibility (inter-assay variation, %CV)	D	No	
Power analysis	D	No	
Statistical methods for result significance	**E**	Yes	Main text: Materials and Methods: Statistics and Data Analysis.
Software (source, version)	**E**	Yes	Main text: Materials and Methods: Statistics and Data Analysis.
Cq or raw data submission using RDML	D	No	Available upon request
